# PD-1^HIGH^ Follicular CD4 T Helper Cell Subsets Residing in Lymph Node Germinal Centers Correlate with B Cell Maturation and IgG Production in Rhesus Macaques

**DOI:** 10.3389/fimmu.2014.00085

**Published:** 2014-03-19

**Authors:** Huanbin Xu, Xiaolei Wang, Andrew A. Lackner, Ronald S. Veazey

**Affiliations:** ^1^Division of Comparative Pathology, Tulane National Primate Research Center, Tulane University School of Medicine, Covington, LA, USA

**Keywords:** follicular CD4 T helper cells, PD-1, B cell, IgG, lymph nodes

## Abstract

CD4+ T follicular helper (TFH) cells guide development and maturation of B cells and are crucial for effective antibody responses. Here we found rhesus macaque TFH cells, defined as CXCR5+CD4 T cells, contain two major populations: PD-1^INT^ and PD-1^HIGH^ cells. Of these, PD-1^HIGH^CD4+ T cells highly co-express ICOS but little CCR7, and reside in lymph node germinal centers (GCs), but not in blood. These cells secrete IL-21 and express transcriptional factor Bcl-6 at higher levels than CXCR5+PD-1^INT^CD4+ T cells. In addition, the frequency of PD-1^HIGH^CD4+ T cells is low in lymph nodes of newborns, but increases with age. Levels of PD-1^HIGH^CD4+ T cells correlate with mature B cells in lymph nodes, and PD-1 blockade in PD-1^HIGH^CD4+ T and B cell co-cultures significantly inhibits IgG production. In summary, PD-1^HIGH^CD4+ T cells residing in GC represent a specific TFH subset that contributes to maturation of B cells and IgG production.

## Introduction

T follicular helper (TFH) cells are essential for the germinal center (GC) reaction, isotype class switching, and antibody affinity maturation. After immunization, effector TFH cells are able to migrate into the GCs, and reside within GCs to form stable contacts with GC B cells, providing direct help to B cells for maturation, and effective antibody responses (1–4). Conversely, the absence of T cell help during priming leads to B cell apoptosis, thereby preventing final B cell differentiation and maturation into antibody-secreting plasma cells (1). In humans and mice, TFH cells express CXCR5 and also ICOS, PD-1 Bcl-6 (B cell lymphoma transcription factor 6), and IL-21, which promote B cell differentiation, maturation, and antibody production (5). However, definitive identification of TFH cell subsets and localization in primates remains unclear, as there is a lack of consensus as to whether T cells that provide help to B cells in extra-follicular/interfollicular sites belong to the TFH lineage (3, 6). Notably, PD-1, a potent T cell inhibitory receptor (7, 8), is highly expressed on GC TFH cells, and contributes to regulation and survival of GC B cells through interaction with its ligand (9), suggesting PD-1 intensity maybe useful for identification and discrimination of functional TFH cell subsets in rhesus macaques.

To validate macaque TFH cells and their function in development and maturation of B cell responses, we compared expression of surface CXCR5, PD-1, ICOS, Bcl-6, and IL-21 secretion in CD4 T cells from lymph nodes of normal macaques, and analyzed the distribution, localization, and frequency of PD-1^HIGH^ TFH cells in newborns and adult animals by polychromatic flow cytometry and confocal microscopy, and correlated levels of PD-1^HIGH^CD4 T cells with B cell subset maturation in lymph nodes. Our results showed that surface CXCR5 could be detected on subsets of CD4 T cells and most B cells in lymph nodes. Notably, CXCR5+CD4+ T cells contained both PD-1^INT^ and PD-1^HIGH^ cell populations, the latter highly expressing ICOS and Bcl-6, but low levels of CCR7. Further, PD-1^HIGH^CD4+ T cells were specifically found within GCs of lymph node follicles and produced IL-21. Numbers of PD-1^HIGH^CD4+ T cells increased in LN with age, and appear to play the key role in maturation and IgG production of B cells through PD-1-dependent pathways. Thus, GC PD-1^HIGH^CD4+ T cells appear to represent the major functional TFH cells of macaques.

## Materials and Methods

### Animals and virus

A total of 42 Indian rhesus macaques (*Macaca mulatta*, RMs), which were negative for SIV, type D retrovirus, and STLV-1 infection were utilized to examine TFH cells in lymph nodes. All animals were housed at the Tulane National Primate Research Center in accordance with the Association for Assessment and Accreditation of Laboratory Animal Care International standards, and were performed in compliance with the U.S. Department of Health and Human Services Guide for the Care and Use of Laboratory Animals. All studies were reviewed and approved by the Tulane University Institutional Animal Care and Use Committee. Of these, 10 animals were newborn or infants (age: 0–6 months) or juvenile (age: 6 months to 3 years) and the rest were adults (>3 years, *n* = 32). All animals were euthanized for tissue collection as controls for other studies.

### Tissue collection and phenotyping

Blood and mesenteric lymph nodes were collected and analyzed by flow cytometry. Peripheral blood mononuclear cells (PBMCs) were prepared from blood, and lymph nodes were obtained within 30 min of euthanasia and processed for immunohistochemistry or cell suspensions for flow cytometry as previously described (7). Briefly, lymphocyte suspensions were prepared by mincing and passing mesenteric portions of lymph node tissues through nylon screens and adjacent pieces were embedded and snap-frozen in optimum cutting temperature compound (OCT) and in formalin. Immunohistochemistry and *in situ* detection of specific lymphocyte subsets in lymph nodes was performed on snap-frozen and formalin-fixed organized lymphoid tissues including the LN, spleen, and gut-associated lymphoid tissues (GALT).

Flow cytometry for surface and intracellular staining was performed using standard protocols (10). Cells were stained with: CD3 (SP34), CD4 (L200), CXCR5 (MU5UBEE, eBiosciences), CD20 (2H7), CD27 (M-T271), IgD (SouthernBiotech), IgG (G18-145), ICOS (C398.4A, BioLegend), PD-1 (EH12.2H7, BioLegend), and LIVE/DEAD fixable aqua dead cell stain kit (Invitrogen, Grand Island, NY, USA). Isotype-matched controls were included in all experiments. All antibodies and reagents were purchased from BD Biosciences Pharmingen (San Diego, CA, USA) unless otherwise noted. Samples were resuspended in BD stabilizing fixative (BD Biosciences) and acquired on a FORTESSA flow cytometer (Becton Dickinson, San Jose, CA, USA). Data were analyzed with FlowJo software (Tree Star, Ashland, OR, USA).

### Multi-color confocal microscopy and immunohistochemistry

Snap-frozen LN were sectioned and stained using unconjugated primary antibodies (CD3, CD4, CD20, and PD-1) followed by appropriate secondary antibodies conjugated to the fluorescent dyes Alexa 488 (green), Alexa 568 (red), or Alexa 633 (blue) (Molecular Probes, Eugene, OR, USA). Confocal microscopy was performed using a Leica TCS SP2 confocal microscope equipped with three lasers (Leica Microsystems, Exton, PA, USA). Individual optical slices representing 0.2 μm and 32–62 optical slices were collected at 512 × 512 pixel resolution. NIH image (version 1.63, Bethesda, MD, USA) and Adobe Photoshop CS5 (San Jose, CA, USA) were used to assign colors to the channels collected. To detect PD-1 expression in lymph nodes by immunohistochemistry, formalin-fixed, paraffin-embedded sections were deparaffinized, and antigens were unmasked using high-temperature antigen retrieval by heating slides in a steam bath chamber (Flavor Scenter Steamer Plus; Black and Decker, Hunt Valley, MD, USA) with 0.01 M citrate buffer pH 6.0 for 20 min. Slides were then cooled, washed twice in phosphate-buffered saline (PBS), and blocked with peroxidase blocking reagent (Dako, Glostrup, Denmark) for 10 min, washed again in PBS, and further blocked with serum-free protein block (Dako) for 30 min. Sections were then incubated with the purified anti-PD-1 Ab for 1 h at room temperature, washed (PBS), and developed using a Vectastain ABC peroxidase kit (Vector Laboratories, Burlingame, CA, USA) and 3,3-diaminobenzidine DAB (Biocare Medical, Concord, CA, USA).

### Cell stimulation for detection of cytokines

Lymphocytes (10^6^) isolated from lymph nodes were stimulated with 0.1 μM phorbol 12-myristate-13-acetate (PMA) and 0.5 μg/ml ionomycin (Sigma-Aldrich, St. Louis, MO, USA) for 4 h in the presence of 5 μg/ml Brefeldin A (Sigma-Aldrich) at 37°C in a humidified CO_2_ incubator. Cells were then stained for CD3, CD4, and PD-1, washed, then fixed and permeabilized in cytofix/cytoperm solution (BD Biosciences), and intracellularly co-stained with anti-IL-21 antibody (3AS-N2, BD Pharmingen), and acquired with a FORTESSA cytometer (Becton Dickinson). Data was analyzed with FlowJo software (Tree Star, Ashland, OR, USA).

### Autologous lymph node PD-1^HIGH^CD4+ T cell and B cell co-cultures

To assess functional roles of PD-1 on PD-1^HIGH^CD4 T cells in B cell maturation and antibody secretion, PD-1^HIGH^CD4 T cells and B cells were positively sorted from mesenteric lymph node cell suspensions using a MicroBead kit (Miltenyi Biotec) and a FACS Aria sorter, and cells were assessed as >95% pure by flow cytometry. Purified B cells (CD20+, 10^5^ cells/well) were cultured either in media alone or with the same number of purified autologous PD-1^HIGH^CD4 T cells in triplicate in 96-well round bottom plates. To evaluate the effects of PD-1 on IgG secretion of B cells, anti-PD-1 (10 μg/ml) or isotype control antibodies were added to co-cultures on day 1. Supernatants were collected after 11 days and analyzed for IgG levels using isotype-specific Abs and an ELISA (Life Diagnostics, PA, USA).

### Statistics

Graphical presentation and statistical analysis of the data were performed using GraphPad Prism 4.0 (GraphPad Software, San Diego, CA, USA). Comparisons between groups were analyzed by a one-way ANOVA and a non-parametric Mann–Whitney *U*-test. *p* Values <0.05 were considered statistically significant. Correlations between samples were calculated using Spearman’s coefficient of correlation.

## Results

### CXCR5+CD4 TFH cells express PD-1^HIGH^ in lymph nodes of rhesus macaques

**Figure 1:**
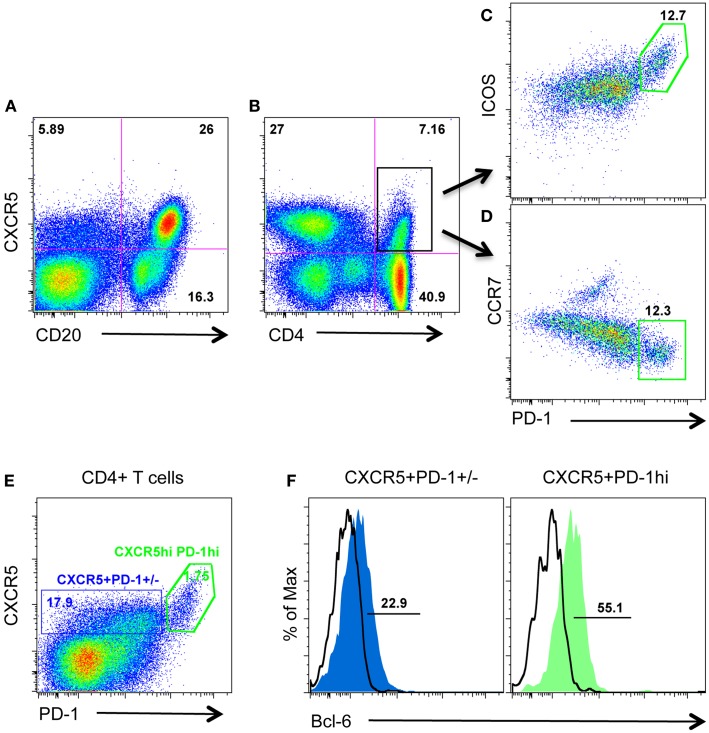
**Surface CXCR5 expression on lymphocytes in lymph nodes of rhesus macaques**. **(A,B)** Representative plots of CXCR5 expression on B cells and CD4+ T cells in lymph nodes from macaques; **(C,D)** representative plots of other TFH cell-associated PD-1, ICOS, and CCR7 expression on CXCR5+CD4+ T cells/TFH cells from lymph nodes; **(E)** CXCR5+CD4 T cells contain both PD-1^INT^ and PD-1^HIGH^ cell populations; **(F)** transcriptional factor Bcl-6 expression in CXCR5+PD-1^INT^ and CXCR5+PD-1^HIGH^CD4 T cell subsets.

CXCR5 is required for migration to the follicular compartment and GC responses, and is typically used to identify TFH cells in human and mice. Further, intracellular CXCR5 mRNA has been detected in PD-1^HIGH^CD4 T cells in lymph nodes from rhesus macaques (11). We thus examined surface CXCR5 protein expression on CD4 T cells from macaque lymph nodes in combination with other known TFH cell-associated molecules including PD-1, ICOS, CCR7, Bcl-6, and IL-21. As shown in Figure 1, surface CXCR5 could be detected on subsets of CD4 T cell as well as the majority of B cells (Figures 1A,B). Further, CXCR5+CD4 T cells (TFH cells) contained two major cell populations: ICOS^INT^PD-1^INT^ and ICOS^HIGH^PD-1^HIGH^ (Figures 1C,D). Interestingly, CXCR5+PD-1^HIGH^CD4+ T cells expressed higher levels of transcriptional factor Bcl-6 and lower levels of CCR7, compared with PD-1^INT^CD4 T cells (Figures 1D,F). These findings suggest PD-1^HIGH^CD4+ T cells represent a distinct subset of an otherwise heterogeneous TFH population in macaque lymph nodes.

### Distribution, localization, and development of PD-1^HIGH^CD4+ TFH cells in rhesus macaques

**Figure 2:**
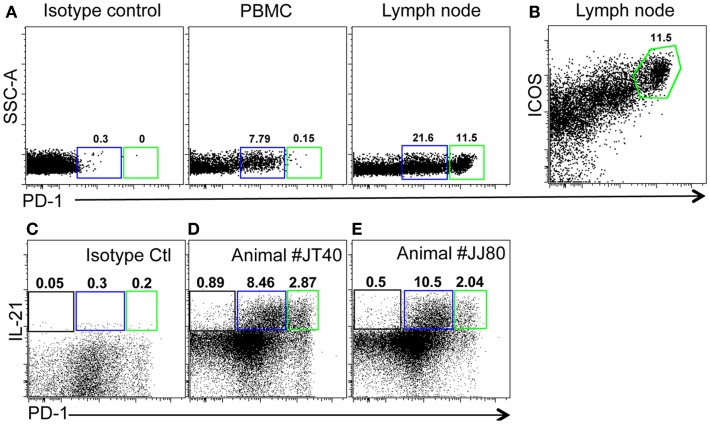
**Phenotyping and features of PD-1^HIGH^CD4+ T cells in blood and mesenteric lymph nodes of adult rhesus macaques**. **(A)** Representative plots of PD-1^HIGH^CD4 T cells in PBMC and lymph node. **(B)** High co-expression of ICOS on PD-1^HIGH^ cells gated through CD4+ cells in lymph nodes. **(C)** Isotype control and IL-21 secretion **(D,E)** of CD4 T cell subsets from two healthy animal (JT40 and JJ80)-derived lymph nodes. Higher IL-21 secretion following mitogen stimulation of both PD-1^INT^ (blue gate) and PD-1^HIGH^ (green gates) by CD4+ T cells from lymph node cell suspensions [**(C,D)**, gated on CD4+ T cells].

**Figure 3:**
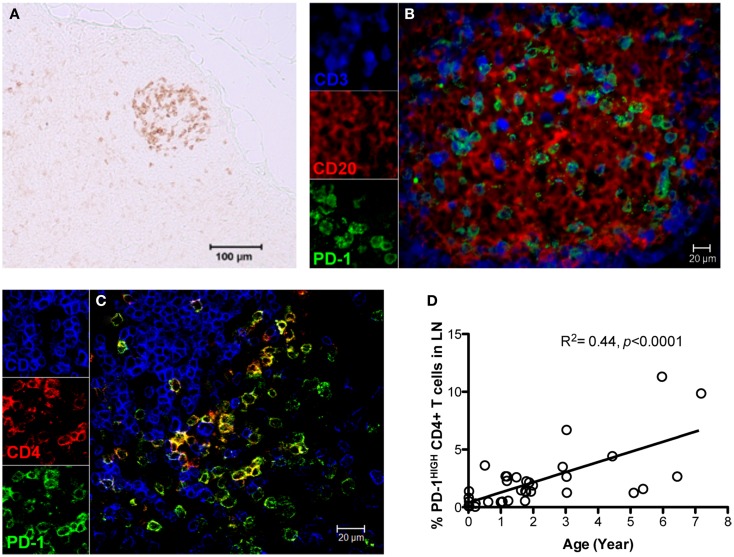
**Distribution and localization of PD-1^HIGH^CD4+ T cells in germinal centers (GCs) of lymph nodes, and their frequency in newborns and juvenile/adult macaques**. **(A)** PD-1 positive cells residing in GC of follicles in lymph nodes of adult macaques detected by immunochemistry (scale bar, 100 μm); **(B,C)** Confocal microscopy analysis of PD-1^HIGH^CD4+ T cells in GC of lymph nodes in adult animals. CD3, blue; PD-1, green; CD20 **(B)** or CD4 **(C)**, red. **(D)** Correlation of PD-1^HIGH^CD4+ T cells with age in rhesus macaques (*R*^2^ = 0.44, *p* < 0.0001). PD-1^HIGH^ cells are specifically localized within GC and predominantly on CD4+ T cells. Scale bar, 100 μm **(A)** or 20 μm **(B,C)**.

As described above, PD-1 is constitutively and highly expressed on GC CD4 TFH cells, and contributes to regulation, maturation, and survival of GC B cells (9). Thus, we examined the distribution of PD-1+CD4+ T cells in lymph nodes by immunohistochemistry and found PD-1^HIGH^CD4+ T cells were specifically distributed in lymph nodes and other organized lymphoid tissues including the spleen and Peyer’s patches (GALT – data not shown), but were very rare in peripheral blood (Figure 2A). These PD-1^HIGH^CD4+ T cells also highly co-expressed ICOS, and were able to secrete IL-21 upon mitogen stimulation (Figures 2B,E). Immunochemistry showed PD-1 was highly expressed on CD4 T cells, which were specifically localized in GCs of follicles, often surrounded by germinal B cells (Figures 3A–C), consistent with previous reports of TFH in mice (12). We also found that frequencies of PD-1^HIGH^CD4+ T cells were very low in lymph nodes of neonatal and infant macaques (<6 months), but increased with age in juvenile and adult macaques (>6 months, *R*^2^ = 0.44, *p* < 0.0001) (Figure 3D), which may partially explain the poor follicular Th cell response, reduced GC reactions, and lower B cell responses to vaccines reported in newborns (13, 14). Notably, as shown in Figure 2C, large numbers of PD-1^INT^ICOS^INT^CD4 T cells in lymph nodes were also able to produce functional IL-21 after activation, yet these cells were primarily found in T cell zones or other regions of follicles. These cells may also be involved in TFH cell-induced B cell differentiation, proliferation, and retention in GC, and may represent a transitional stage of TFH cells that are migrating toward, or away from the GC (12, 15, 16). Nonetheless, these findings suggest that PD-1^HIGH^CD4+ T cells represent a specific subset of a heterogeneous population of TFH in rhesus macaques that directly interact with B cells in follicular GC.

### PD-1 on TFH cells mediates B cell maturation and IgG production

**Figure 4:**
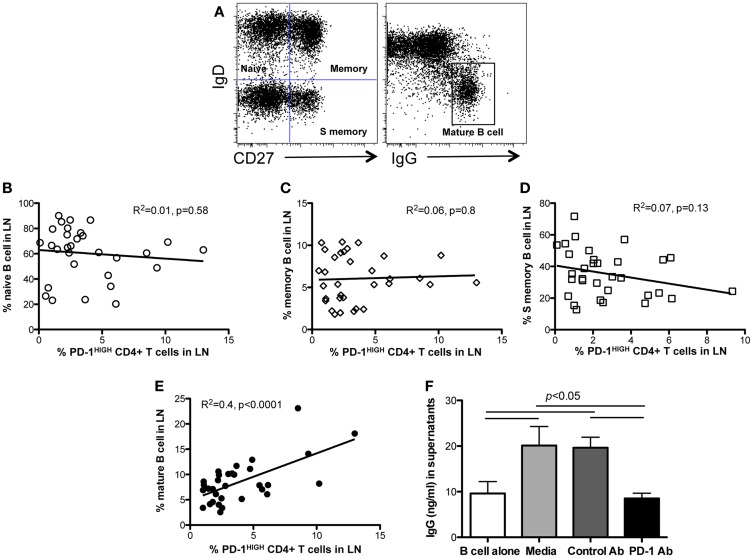
**Correlation of PD-1^HIGH^CD4 T cells with B cell subsets in juvenile/adult macaques, and effects of PD-1 blockade on antibody production in autologous LN-derived PD-1^HIGH^CD4 T/B cells co-cultures**. **(A)** Dot plot showing gating strategy for defining B cell subsets in lymph nodes. Naïve (IgD+CD27−), memory (IgD+CD27+), switched memory (IgD−CD27+), and mature (IgD−IgG+) B cell subsets are distinguishable; **(B–E)** correlation of PD-1^HIGH^CD4 T cells with B cell subsets in juvenile/adult macaques. Note that PD-1^HIGH^CD4 T cells positively correlate with mature B cells in LNs, but not naïve, memory, or switched memory B cells. **(F)** IgG production in autologous PD-1^HIGH^CD4+ T cell and B cell co-cultures after PD-1 blockade. Supernatants were collected 11 days after culture and IgG levels detected by ELISA. Note that PD-1 blockade significantly inhibited IgG production in co-culture *in vitro*. The data in **(F)** show results from five different normal adult animals, and all experiments were performed in triplicate. *Indicates *p* < 0.05.

To investigate the effects of PD-1^HIGH^CD4 T cells on B cell differentiation and maturation, we compared B cell subsets in lymph nodes of juvenile and adult macaques (age >6 months). Neither naïve B cells (CD27−IgD+), memory B cells (CD27+IgD+) nor “switched” memory B cells (CD27+IgD−) significantly correlated with levels of PD-1^HIGH^CD4 T cells in mesenteric lymph nodes (Figures 4A–D). In contrast, percentages of mature B cells (IgD−IgG+) positively correlated with PD-1^HIGH^CD4 T cells (*R*^2^ = 0.4, *p* < 0.0001) (Figure 4E), suggesting PD-1^HIGH^CD4 T cells in GCs are involved in maturation of B cells.

Although PD-1 expression is associated with inhibition of T-cell responses in chronic viral infections (7, 17), high PD-1 expression on TFH cells is also critical for regulating GC formation, B cell survival, affinity maturation of plasma cells, and IgA secretion in the gut (9, 18). We thus performed *in vitro* blockade experiments with anti-PD-1 antibody in autologous lymph node-derived B and PD-1^HIGH^CD4 T cell co-cultures (without mitogen stimulation) for up to 11 days. The results showed that B cells alone produce low levels of IgG constitutively, yet CD4 T and B cell co-cultures had significantly higher IgG production. Interestingly, PD-1 blockade significantly reduced IgG production (Figure 4F). Thus PD-1, which is highly expressed on TFH cells, appears to play an important role in promoting IgG antibody production in CD4+ T and B cell interactions in lymph nodes.

## Discussion

T follicular helper cells are a specialized subset of effector T cells that provide the required signals for GC reactions and high-affinity maturation of B cells. Here we show surface CXCR5 is expressed on CD4+ T cell subsets and the majority of B cells in lymph nodes of macaques. As TFH cells, CXCR5+ CD4 T cells contain both PD-1^INT^ and PD-1^HIGH^CD4 T cells, and the latter co-express higher levels ICOS and Bcl-6. These PD-1^HIGH^CD4 T cells are able to produce IL-21 after activation, and are predominantly distributed in GCs of follicles in lymph nodes, but not CD4 T cells derived from peripheral blood. However, considerable numbers of extra-follicular PD-1^int^ICOS^int^ CD4 T cells are distributed in T cell zones and marginal areas of follicles surrounding GC and these also have ability to produce IL-21, consistent with reports in mice (12). Moreover, neonates and infants (<6 months) have significantly lower frequencies of PD-1^HIGH^ TFH cells in lymph nodes, which gradually increase with age, which may explain suboptimal responses of infants to vaccination. Finally, PD-1^HIGH^CD4 T cells positively correlate with mature B cells, but not naïve, memory B, or switched B memory cells in lymph nodes in juvenile and adult macaques, and PD-1 blockade significantly inhibits IgG production in autologous B and PD-1^HIGH^CD4 T cell co-cultures *in vitro*. These findings suggest TFH maturation, and possibly migration patterns develop with age, and that eventually it is the PD-1^HIGH^CD4 T cell subset of TFH cells that plays the ultimate role in directing B cell maturation and effective humoral immune responses.

T follicular helper cells are critical to multiple steps in B cell maturation and differentiation within follicular GCs. GCs, as specialized structures in follicles of secondary lymphoid tissues including lymph nodes, spleen, tonsils, and the Peyer’s patches of GALT, are the crucial niche for the optimal expansion and survival of activated T cells (19, 20), and processes such as somatic hypermutation, class switch recombination, and selection of high-affinity B cells (21, 22). CXCR5, a key surface marker of TFH cells, is involved in homing of TFH cells to the CXCL13-rich B cell follicles and T cell-dependent humoral responses. It was recently reported that PD-1^HIGH^CD4+ T cells in lymph nodes of rhesus macaques contain CXCR5 mRNA (11). Here we report that surface CXCR5 is detectable on both CD4 and B cells and on both PD-1^HIGH^ and PD-1^INT^ CD4 T cells in lymph nodes. However, our immunohistochemistry data show that PD-1^HIGH^CD4+ T cells specifically reside in the GC of follicles in the GALT and other secondary lymphoid tissues (spleen), but not in peripheral blood (Figures 2 and 3), and concomitantly co-express ICOS, Bcl-6, IL-21, and have low expression of CCR7 and CD62L (data now shown), consistent with descriptions of TFH cells in humans and mice (8, 23). Interestingly, the frequencies of PD-1^HIGH^CD4+ T cells were observed low level in lymph nodes of neonatal and infant macaques (<6 months), and increased with age (Figure 3D). It is reported that vaccines elicit lower GC reaction and low level of antibody responses in neonates (13, 14), which could be attributed to limited number follicular TFH cells in newborns. Thus, the characteristic of TFH cells in newborns may provide clue for appropriate vaccine strategy. For example, the HBV vaccine is generally given on a schedule of 0, 1, and 6 months after birth (24), it is thus speculated that best window of effective HBV vaccine immunization is probably at 6 months and later. It has also been shown that PD-1^HIGH^CD4 T cells in GCs are most efficient for providing B cell help by producing IL-21 (12). Notably, our results show that some PD-1^INT^CD4 T cells in lymph nodes also produce IL-21 after activation. Further, due to PD-1^INT^CD4 T cells present mainly in the T cell area or other region in lymph nodes (12), the PD-1^INT^CD4 T cells expressing CXCR5 are often lumped with other TFH cells when examining cell suspensions by flow cytometry. These IL-21+ PD-1^INT^CD4 T cells may also be involved in B cell differentiation, and may represent a transitional stage of TFH cells that are migrating toward, or away from the GC (15, 16). Here we show, PD-1^HIGH^CD4+ T cells represent a specific, highly functional subset of TFH cells that are restricted to GC of follicles, where they direct B cell maturation.

Since PD-1 is highly expressed on GC TFH cells, this is likely key to its interaction with its ligands expressed by GC B cells, which may contribute to their regulation and survival (9). However, PD-1 is also a potent inhibitory receptor for T cells, and is associated with T-cell “exhaustion” or suppression (7, 8, 25). As shown in Figure 4, PD-1^HIGH^CD4+ T cells positively correlate with mature B cells, but not naïve and memory B cells, and constitutive, high expression of PD-1 on CD4+ T cells from lymph nodes promotes IgG production in B/T cell co-cultures, and these effects are markedly reduced by PD-1 blockade. This fine-tuned regulation between CD4+ T cells and B cells in lymphoid tissues may prove important for discovering mechanisms associated with adaptive immune responses that should be considered when designing immunotherapy strategies, such as PD-1 blockade.

In summary, PD-1^HIGH^CD4+ T cells express CXCR5, ICOS^HIGH^, Bcl-6, and IL-21, and represent a specific highly functional subset of TFH cells in organized lymphoid tissues of rhesus macaques. These specific subsets are rare in newborns, who classically display poor or delayed B cell responses that increase with age. High PD-1 expression on specific TFH cell subsets may thus play an important role in promoting antibody production through direct interaction with its ligand on B cells within GC, and these findings provide clues for designing more effective vaccination and therapeutic strategies.

## Conflict of Interest Statement

The authors declare that the research was conducted in the absence of any commercial or financial relationships that could be construed as a potential conflict of interest.
